# Transgene-free genome editing in citrus and poplar meristem tissues via biolistic ribonucleoprotein delivery of CRISPR-Cas9

**DOI:** 10.1007/s00299-026-03741-9

**Published:** 2026-02-17

**Authors:** Dhiôvanna Corrêia Rocha, Miracle Osazee Omoregbee, Weifeng Luo, Hong Fang, Qiushi Ye, Yanxin Liu, Gen Li, Juliet Mascoveto, Alessandra Alves de Souza, Gary Coleman, James N. Culver, Yiping Qi

**Affiliations:** 1 Department of Plant Science and Landscape Architecture, University of Maryland, College Park, MD USA; 2 Citrus Research Center ‘Sylvio Moreira’-Agronomic Institute (IAC), Cordeirópolis, SP Brazil; 3 Institute of Biology, State University of Campinas (UNICAMP), Campinas, SP Brazil; 4 Department of Chemistry and Biochemistry, University of Maryland, College Park, MD USA; 5 Institute of Bioscience and Biotechnology Research, University of Maryland, Rockville, MD USA

**Keywords:** Biolistic, CRISPR-Cas9, RNP-delivery, Shoot apical meristem, Axillary meristem, Trees

## Abstract

**Key message:**

Biolistic particle bombardment was used to deliver CRISPR-Cas9 ribonucleoprotein complexes (RNP) into the shoot apical meristem tissue of citrus and axillary meristem tissue of poplar, generating directed mutations in target genes.

**Abstract:**

The use of meristematic tissues offers a strategic approach to genome editing in woody species, especially those that are recalcitrant to conventional tissue culture, as these regions contain totipotent, highly regenerative cells capable of giving rise to whole plants. Here, we employed biolistic delivery of genome-editing reagents into theshoot apical meristem (SAM) of citrus and the axillary meristems (AXM) of poplar. The system was first validated using a GFP expression construct and subsequently applied for targeted genome editing. In citrus, edited plants were obtained at the CsNPR3 locus exclusively through the delivery of CRISPR/Cas9 ribonucleoproteins (RNPs), whereas plasmid-based vectors were unsuccessful. Similarly, genome editing in poplar was achieved using RNPs targeting the Pt4CL1 gene. Although chimeric events were detected, this approach provides a feasible and innovative framework for producing transgene-free edited perennial plants.

**Supplementary Information:**

The online version contains supplementary material available at 10.1007/s00299-026-03741-9.

Plant meristems are tissue composed of undifferentiated cells that preserve the capacity to generate all cell types, thereby maintaining the plant’s regenerative potential throughout its lifespan (Kean-Galeno et al. 2024; Gaillochet and Lohmann 2015). These regions are essential for plant development and sustained growth, enabling continuous organ formation, adaptation to changing environmental conditions, and recovery from damage. The shoot apical meristem (SAM) and axillary meristems (AXM) play central roles in shaping above-ground plant development and architecture. Within the SAM and AXM, L2 sub-epidermal cells retain the potential to form germ cells, so introducing heritable mutations into these cells enables the generation of stably edited plants (Kean-Galeno et al. 2024; Gaillochet and Lohmann 2015).This characteristic make SAM and AXM tissue an appealing target for genome-editing approaches aimed at generating modified plants, especially in recalcitrant species that experience significant regeneration constraints in tissue culture.

Genome editing using the SAM and AXM as target tissues has been successfully demonstrated for multiple plant species using different transformation methods that has resulted in efficient plant regeneration (Lian et al. 2022; Luo et al. 2023; Maher et al. 2020). Meristem explants, when used in combination with particle bombardment, have enabled the production of transgene-free edited wheat, melon, barley, and soybean (Kumagai et al. 2022; Kuwabara et al. 2024; Sasaki et al. 2025; Tezuka et al. 2024), which can facilitate the regulatory approval process and the commercial release of genetically modified plants. Consequently, such methods hold great promise for generating transgene-free genome-edited perennial trees.

In this study, we explored the biolistic method to deliver genome-editing reagents into meristematic tissues, aiming to generate transgene-free, gene-edited citrus and poplar plants. For this purpose, the SAM was exposed from mature citrus seeds (Fig. 1a and Supplementary Fig. 1a) and the AXM of in vitro grown poplar branch plantlets were used as explants (Supplementary Fig. 2a).

**Fig. 1:**
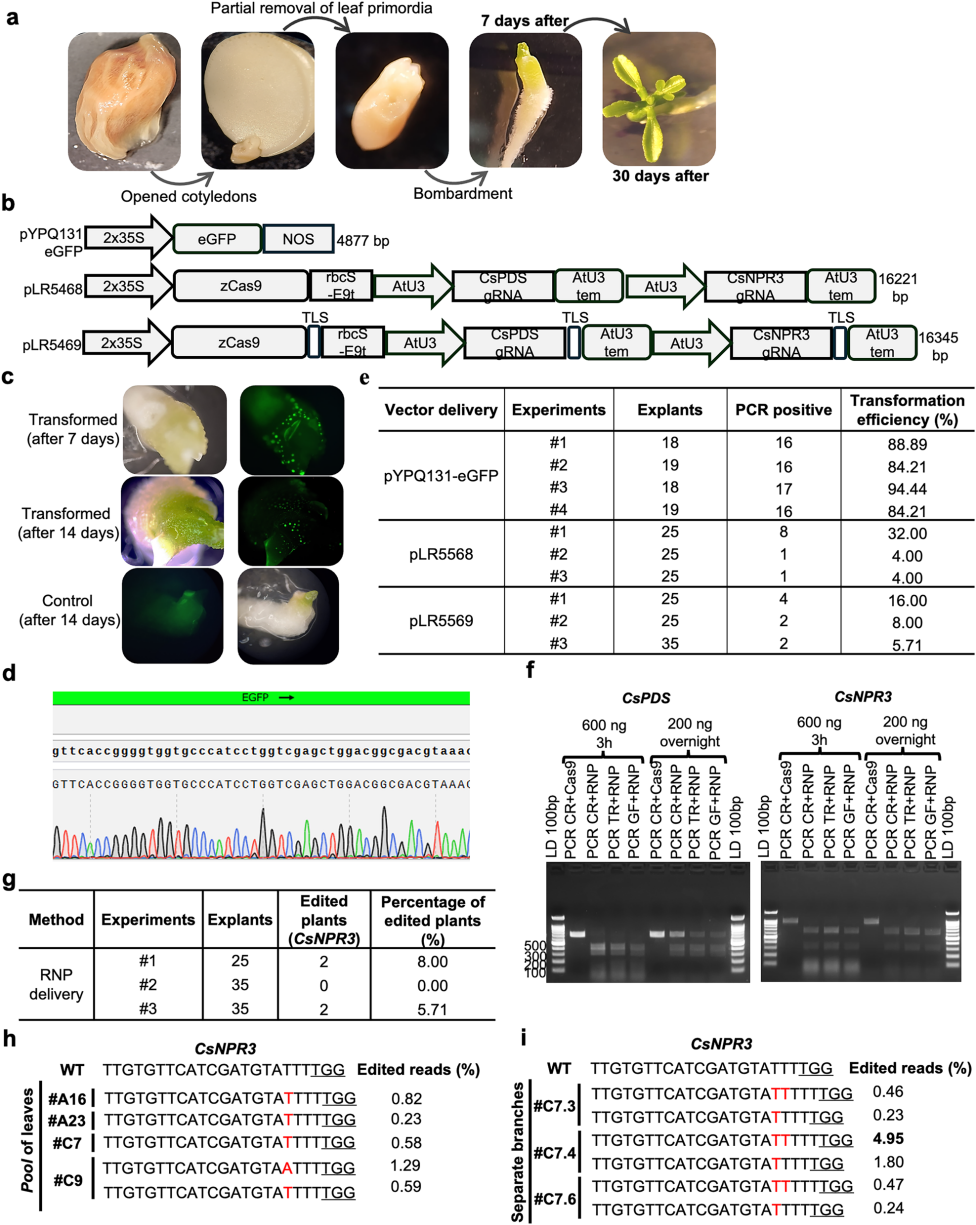
Transformation of citrus shoot apical meristems (SAMs) by biolistic delivery of plasmid vectors. **a** SAM preparation for the biolistic experiments. **b** Vector maps used for expression of reporter gene (pYPQ131-eGPF) and for genome editing (pLR5468 and pLR5469). **c** Example of fluorescence observed in explants successfully transformed with the vector pYQP131-eGFP. **d** Sanger sequencing performed to confirm the presence of the GFP sequence in fluorescent shoots. **e** Transformation efficiency calculated based on the initial number of explants and the PCR-positive shoots for each experiment. **f** Cleavage assay to test the activity of the sgRNAs in vitro prior to RNP bombardment. *LD:* 100 bp DNA ladders; *PCR CR:* PCR performed using Carrizo DNA; *PCR TR:* PCR performed using Troyer DNA; *PCR GF:* PCR performed using Grapefruit DNA. **g** Percentage of edited plants from RNP delivery. **h** Sequencing of shoots using DNA extracted from a pooled sample of leaves. **i** Identification and characterization of edited branches

First, the particle bombardment parameters were tested using the pYPQ131-eGFP vector (Fig. 1b), which contains a Green Fluorescent Protein (GFP) expression cassette that allows easy visualization of transformed tissues and cells. To validate the success of the genetic transformation, after fluorescence detection (Fig. 1c), genomic DNA was extracted and a fragment of the GFP vector was amplified by PCR (Supplementary Fig. 1b), and five randomly selected samples were subjected to Sanger sequencing (Fig. 1d). As a result, bombarded citrus explants showed a remarkably high average transformation efficiency of 87.9% (Fig. 1e), while poplar exhibited an average efficiency of 13% for three experiments (Supplementary Fig. 2b and 2c). Additionally, the regeneration time of explants derived from the citrus SAM was demonstrated to be remarkably rapid and within 30 days it is already possible to obtain a well-developed seedling, including both aerial and root structures (Fig. 1a).

After the genetic transformation with the GFP construct, additional transformations were carried out in citrus using two vectors, pLR5468 and pLR5469 (Fig. 1b), containing components for genome editing of two citrus genes at the same time: *CsPDS (ID: Cs_ont_9g016420, C. sinensis* v3.0), which generates an albino phenotype when knocked out, and *CsNPR3* (ID: Cs_ont_2g009270, *C. sinensis* v3.0), a gene associated with disease tolerance in citrus. Following plant regeneration, DNA was extracted from leaf tissue and PCR was performed to identify transgenic shoots (Supplementary Fig. 1c). pLR5469 differs from pLR5468 in that both Cas9 and gRNA are tagged with the mobile RNA sequence, TLS2 (Yang et al. 2023). The average transformation efficiencies obtained were 13.33% and 9.90% for vectors pLR5468 and pLR5469, respectively (Fig. 1e). However, no albino phenotype was observed among the regenerated shoots, indicating that *CsPDS* was not edited in both alleles (Supplementary Fig. 3a). Thus, to assess potential genome-editing events, the *CsNPR3* gene was analyzed using next-generation sequencing (NGS) of PCR amplicons, but no mutations were detected in this gene either (Supplementary Fig. 3b).

Therefore, the transformation efficiency obtained with the genome-editing vectors was lower than that achieved with the GFP construct, likely due to the size difference among the constructs. Successful genome editing depends on all editing components of CRISPR-Cas9 being delivered intact and functional. Given that biolistic delivery can cause DNA fragmentation and molecular instability, our results suggest the editing system may not have been fully or properly delivered, which could have prevented genome editing from occurring. We reasoned that delivering CRISPR-Cas9 in the form of ribonucleoprotein complex (RNP) could not only address this problem but also present a DNA-free genome editing approach, which is highly valuable in perennial trees as segregation of transgenes is not a feasible option.

To this end, we constructed a Cas9 vector tailored for *E. coli* expression (Supplementary Fig. 4a) and purified the Cas9 protein (Supplementary Fig. 4b). In vitro cleavage assays using the sgRNAs targeting the genes of interest were conducted before the bombardment experiments. For citrus, the sgRNA targeting the *CsNPR3* gene showed higher cleavage efficiency than the sgRNA targeting *CsPDS*, as it was able to completely cleave the target fragment in both tested conditions (Fig. 1f). Although the *CsPDS* sgRNA was less efficient, both sgRNAs were used simultaneously in the RNP particle bombardment experiments.

After shoot development, no albino phenotype was observed in the citrus plants, based on three independent experiments (Fig. 1g). Therefore, only the *CsNPR3* target site was analyzed by sequencing. Initially, DNA was extracted from a pooled leaf sample and four plants showed T or A single nucleotide insertions at the cleavage site (Fig. 1g), and up to 1.29% editing efficiency was detected for one shoot (Fig. 1h). To determine which branches within these positive shoots were edited, DNA was subsequently extracted from individual leaves. This analysis confirmed the chimeric edited branches and revealed that one branch has an editing efficiency of 4.95% (Fig. 1i).

For poplar, the sgRNA targeting *PtPDS* (ID: PtXaTreH.14G119600.1 × PtXaAlbH.14G121300.1) gene did not efficiently cleave the target fragment in the in vitro cleavage assay, whereas the sgRNA targeting *Pt4CL1* (ID: PtXaTreH.01G031400x PtXaAlbH.01G031600) produced a clear cleavage product (Supplementary Fig. 5a). Therefore, only the *Pt4CL1* sgRNA was used for the poplar bombardment experiments. As a result, six plants displaying chimeric editing were obtained, containing A or C insertions as well as single-nucleotide deletions at the cleavage site, and in one line (#8–17), the editing efficieny within the individual shoot efficiency was over 1% (Supplementary Fig. 5b and 5c), indicating only a small fraction of the cells were edited.

In summary, we applied particle bombardment to target the SAM tissue and AXM tissue in citrus and poplar, respectively, enabling the generation of transgenic plants in these species, albeit with chimerism. We also show that this approach allows the production of transgene-free edited chimeric citrus and poplar plants through RNP delivery, but not through DNA delivery. Our data point to a promising direction for further optimization of the protocols to reduce chimerism and achieve higher editing efficiencies. As continuous shoot cutting and enrichment is a traditional practice to fix chimeric mutations and phenotypes in trees, we believe that our success in generating chimeric mutations with RNP delivery of CRISPR-Cas9 provides a path to the generation of genome-edited trees with the target gene fully edited.

## Supplementary Information

Below is the link to the electronic supplementary material.

Supplementary file1 (DOCX 1123 KB)

## Data Availability

All our data were included in the main figure and supplementary information. Request for the raw data should be communicated to the corresponding author.
