# Supplementary material for: Transgene-free genome editing in citrus and poplar meristem tissues via biolistic ribonucleoprotein delivery of CRISPR-Cas9
Source: Plant Cell Rep. 2026 Feb 17;45(3):58. doi: 10.1007/s00299-026-03741-9 (PMC12913300; doi:10.1007/s00299-026-03741-9)
Supplement: Supplementary file 1 — Supplementary file1 (DOCX 1123 KB) [file 299_2026_3741_MOESM1_ESM.docx]

*Research notes*

**Transgene-free genome editing in citrus and poplar meristem tissues via biolistic ribonucleoprotein delivery of CRISPR-Cas9**

Dhiôvanna Corrêia Rocha^1,2,3^, Miracle Osazee Omoregbee^1^, Weifeng Luo^1^, Hong Fang^1^, Qiushi Ye^4,5^, Yanxin Liu^4,5^, Gen Li^1^, Juliet Mascoveto^1^, Alessandra Alves de Souza^2^, Gary Coleman^1,5^, James N. Culver^1,5^, Yiping Qi^1,5,*^

^1^Department of Plant Science and Landscape Architecture, University of Maryland, College Park, MD, USA.

^2^Citrus Research Center ‘Sylvio Moreira’-Agronomic Institute (IAC), Cordeirópolis, SP, Brazil

^3^Institute of Biology, State University of Campinas (UNICAMP), Campinas, SP, Brazil.

^4^Department of Chemistry and Biochemistry, University of Maryland, College Park, MD, USA.

^5^Institute of Bioscience and Biotechnology Research, University of Maryland, Rockville, MD, USA.

**^*^Corresponding author:** Yiping Qi ([yiping@umd.edu](mailto:yiping@umd.edu))

**SUPPLEMENTARY METHODS**

**Citrus shoot apical meristem (SAM) preparation**

Mature Carrizo seeds (from Lyn Citrus Seeds, Inc) were sterilized for 15 minutes with sodium hypochlorite (20%), washed 3 times, and then kept for overnight at 4ºC. Subsequently, using a stereo microscope (Nikon SMZ745T), the cotyledons were opened, and the embryo removed. To expose the SAM region, leaf primordia were partially excised, and the explant was placed on MS medium supplemented with Plant Preservative Mixture (PPM 3%; Nacalai Tesque, Japan) and phytagel (6.0 g/L; Sigma-Aldrich). Embryos were bombarded separately with the pYQP131-eGFP, pLR5568, pLR5569 vectors, and with CRISPR-Cas9 RNPs for transgene-free genome editing.

**Poplar axillary meristem (AXM) preparation**

*Populus alba X Populus tremula* 717 IB4 plantlets were grown on LS media (Linsmaier and Skoog, 1965) containing 0.1 mg/L IBA at 23ºC with continuous lighting at an intensity of approximately 100 µmol/m^2^/s. Rooted plantlets approximately 2 months old were used for axillary meristem bombardment. Since the axillary meristem of poplar is typically enclosed by five bud scales (Rinne *et al.*, 2015), the axillary meristem was exposed by dissection and removal of both the bud scales and embryonic leaves, and the shoot explants were placed on LS media containing 0.1 mg/L IBA and PPM 1.5%. After maintaining the explants in the dark for four days, shoots with exposed axillary meristems were bombarded with the pYQP131-eGFP vector and, separately, with CRISPR-Cas9 RNPs for transgene-free genome editing.

**Vector assembly for genome editing in citrus**

The corresponding oligonucleotides for each sgRNA were designed (Supplementary Tables 1 and 3), synthesized, annealed, and ligated into the vectors pYPQ131 (Addgene #69281) and pYPQ132 (Addgene #69282) at BsmBI (Esp3I) restriction sites using T4 DNA ligase, following our previous protocol (Lowder et al., 2015). The *CsPDS* sgRNA used was previously reported for citrus by others (Tang et al., 2021). To generate a multiplexed sgRNA expression system, a Golden Gate reaction was performed using BsaI-HF and T4 ligase, producing the construct pYPQ142_CsPDS_CsNPR3. Finally, a Multisite Gateway LR reaction (LR Clonase II, Invitrogen) was conducted to assemble the final T-DNA vectors. The entry vectors used in this reaction were pYPQ142_CsPDS_CsNPR3 and pYPQ166 (Addgene #109328), and the destination vector was pCGS710. The resulting final vector was designated pLR5468. For the assembly of second vector, named pLR5469, the same procedure was followed. However, prior to the steps described above, the mobile RNA TLS2 sequence was synthesized as a gene fragment by Integrated DNA Technologies (IDT) and inserted into the vectors pYPQ131B, pYPQ132B, and pYPQ166 via a HiFi assembly reaction (NEBuilder HiFi DNA Assembly Master Mix, New England Biolabs, Ipswich, MA, USA). The primers used for the assembly of pLR5469 are listed in Supplementary Table 1.

**Preparation of microprojectiles for vector bombardment**

For the transformation experiments, 5 μg of vector DNA were mixed with 10 μL of gold particles (0.6 μM, 100 mg/mL, Bio-Rad), 25 μL of 2.5 M CaCl₂, and 10 μL of 0.1 M spermidine, incubated at room temperature for 10 minutes, and centrifuged at 3,000 rpm for 10 seconds. After washing with 75% ethanol and resuspension in 28 μL of 100% ethanol using a sonicator (1510 Branson), each sample was divided into three macrocarriers. Each petri dish containing explants received three shots using the PDS1000/He particle bombardment device (Bio-Rad) with a 6 cm macrocarrier flight distance. Helium pressure was set to 1,350 psi, and the chamber vacuum was adjusted to 26 inches of Hg.

**Genetic transformation of citrus and poplar plants using the GFP vector**

The GFP vector (pYPQ131-eGFP in which eGFP was driven by a 2x35S promoter) was used. Fluorescence in citrus explants was monitored 7 and 14 days after bombardment using a ZEISS microscope with a GFP filter (excitation 470/40 nm, emission 525/50 nm). GFP-bombarded shoots were then maintained under a 16-hour light / 8-hour dark photoperiod at 24 °C, with subcultures every 15 days. DNA was extracted using the Dellaporta method (Dellaporta et al.,1983), followed by PCR with the primers 131GFP-F3 and 131GFP-R3 (Table 1)**.** Five PCR-positive shoots were sequenced by Sanger sequencing to confirm the presence of the GFP cassette. For poplar, the same vector was used. GFP fluorescence was observed after 24 hours of bombardment using the same microscope. DNA extraction was done using the CTAB method (Doyle 1991).

**In vitro cleavage assay**

Each citrus target region was amplified by PCR using Q5 High-Fidelity DNA polymerase and genomic DNA from Carrizo, Troyer, and Grapefruit. Three microliters of the PCR product were used per cleavage reaction. Chemically modified crRNAs targeting *CsPDS* and *CsNPR3*, along with tracrRNA, were synthesized by IDT. Two *in vitro* cleavage conditions were tested: 1) 200 ng of crRNA, tracrRNA, and Cas9 incubated overnight at 37 °C; and 2) 600 ng of the same components incubated for 3 hours at 37 °C. Reactions were terminated by heating at 65 °C for 20 minutes. For poplar, the condition one was used, with the difference that the crRNA was designed to target the *PtPDS* and *Pt4Cl1* genes.

**Expression and purification of Cas9 protein**

To generate the Cas9 expression construct, the zCas9 coding sequence, including its NLS elements, was isolated from pYPQ166 (Addgene #109328) via SacI and NcoI restriction digestion. The fragment was subsequently ligated into a modified pET27b(+) expression vector at the corresponding restriction sites, thereby adding an N-terminal 6×His tag, a TEV cleavage site and a 5×GS linker to the zCas9 protein. *E. coli* Rosetta (DE3) cells were transformed using this plasmid and grown in Lysogeny Broth (LB) media under Kanamycin antibiotic selection. Cultures were incubated at 37°C until reaching an OD₆₀₀ of 0.5–0.6, then cooled down to 16°C and induced with 0.3 mM isopropyl-β-D-thiogalactoside (IPTG) for protein expression overnight. Cells were harvested by centrifugation, resuspended in lysis buffer with 40 mM Tris-HCl pH 7.5, 480 mM KCl, 2 mM β-mercaptoethanol (βME), and 20 mM imidazole, and then lysed by sonication. The lysate was centrifuged, and the supernatant was subjected to Ni^2+^-NTA affinity chromatography. The elution buffer contained 40 mM Tris-HCl pH 7.5, 150 mM KCl, 1 mM βME, and 300 mM imidazole. During overnight dialysis, the 6×His tag was cleaved by TEV protease and imidazole were removed. The dialysis buffer contained 20 mM Tris-HCl pH 7.5, 150 mM KCl, and 1 mM DTT. Further purification was performed using size-exclusion chromatography (Cytiva HiLoad 16/600 Superdex 200 column). The buffer used for size-exclusion chromatography contained 40 mM HEPES-KOH pH 7.5, 150 mM KCl, and 0.5 mM TCEP. Cas9 eluted at ~66 mL, consistent with a monomer species. Protein purity was confirmed by SDS-PAGE, and concentration was determined by absorbance at 280 nm.

**Preparation of microprojectiles for RNP bombardment**

For RNP delivery in citrus, 100 µM of crRNA for both genes (*CsPDS* and *CsNPR3*), 200 µM of tracrRNA, and 100 µM of Cas9 were added to a solution containing 10x CutSmart buffer (NEB) and DEPC treated H₂O. Both the crRNA and tracrRNA were synthesized by IDT. After incubation at room temperature for 10 minutes, 5 µL of TransIT®-LT1 Transfection Reagent (Sigma) and 9 µL of gold particles (100 mg/mL) were added. The mixture was incubated again at room temperature for 10 minutes. It was then centrifuged at 3,000 rpm for 10 seconds, the supernatant was removed, and the sample was washed with 50 µL of autoclaved Milli-Q water, followed by another centrifugation. Finally, the sample was resuspended in 45 µL of Milli-Q water using a sonicator. Fifteen microliters were applied to each macrocarrier, which were allowed to dry for 1 hour. Each plate containing SAM explants was bombarded three times under the same conditions used for vector delivery, as described above. For RNP delivery in poplar, the RNP complex was assembled by combining 185 µM Cas9, 270 µM tracrRNA, and 270 µM crRNA targeting the *Pt4CL1* gene. The subsequent procedures were carried out following the same protocol described for citrus.

**Next generation sequencing and mutation analysis**

Genome editing was analyzed by next-generation sequencing (NGS) of PCR amplicons using the Illumina HiSeq2500 platform. The primer-barcode system described by Liu et al. (2019), known as Hi-TOM (High-throughput Tracking of Mutations), was employed, and the primers used for the first PCR round are listed in Supplementary Tables 1 and 2. All PCR reactions were performed using the high-fidelity Q5 DNA polymerase (New England Biolabs, Ipswich, MA, USA). Samples were submitted for sequencing to Genewiz (South Plainfield, NJ, USA). Bioinformatic analyses were conducted as described by Rocha D.C. (2025).

**SUPPLEMENTAL FIGURES**


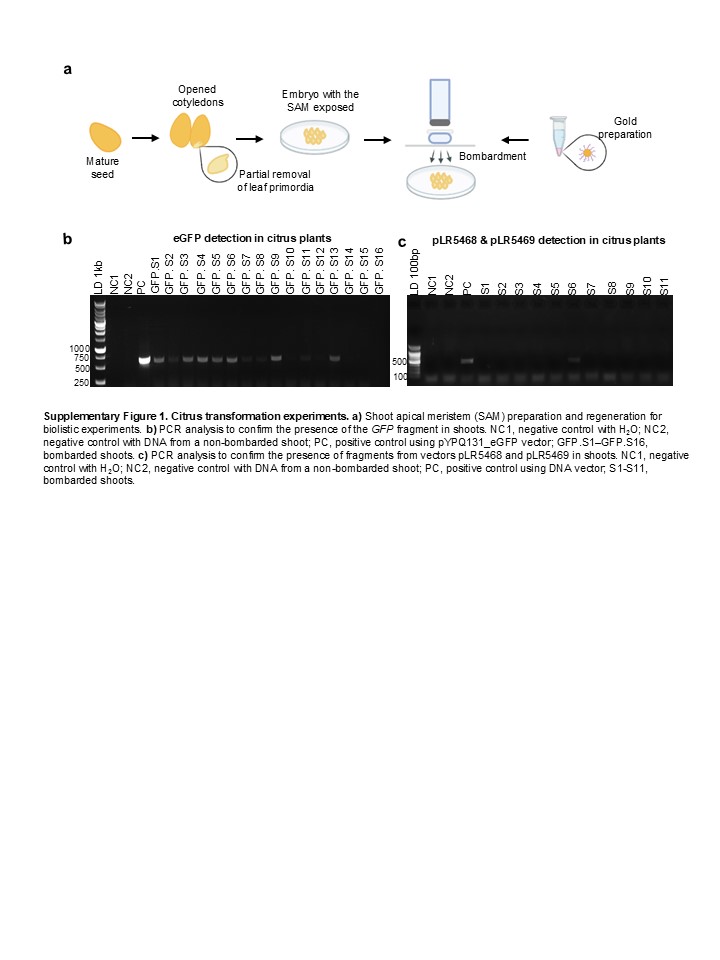


**Supplementary Fig 1**. **Citrus transformation experiments. (a)** Shoot apical meristem (SAM) preparation and regeneration for biolistic experiments. (**b)** PCR analysis to confirm the presence of the *GFP* fragment in shoots. NC1, negative control with H₂O; NC2, negative control with DNA from a non-bombarded shoot; PC, positive control using pYPQ131_eGFP vector; GFP.S1–GFP.S16, bombarded shoots. (**c)** PCR analysis to confirm the presence of fragments from vectors pLR5468 and pLR5469 in shoots. NC1, negative control with H₂O; NC2, negative control with DNA from a non-bombarded shoot; PC, positive control using DNA vector; S1-S11, bombarded shoots.

**
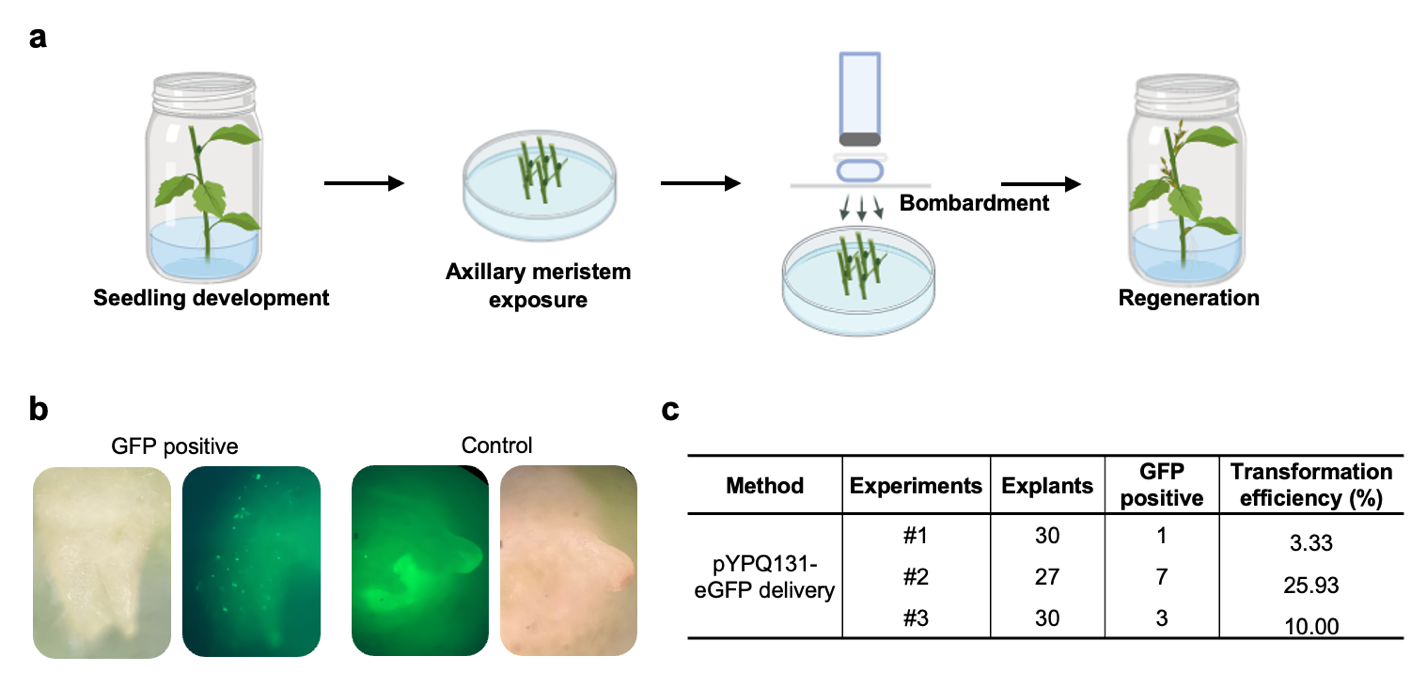
**

**Supplementary Fig 2**. **Poplar transformation experiments.** **(a)** Axillary meristem (AXM) preparation and regeneration for biolistic experiments. **(b)** Example of GFP fluorescence observed in successfully transformed explants. **(c)** Transformation efficiency was calculated based on the initial number of explants and the GFP -positive shoots for each experiment.


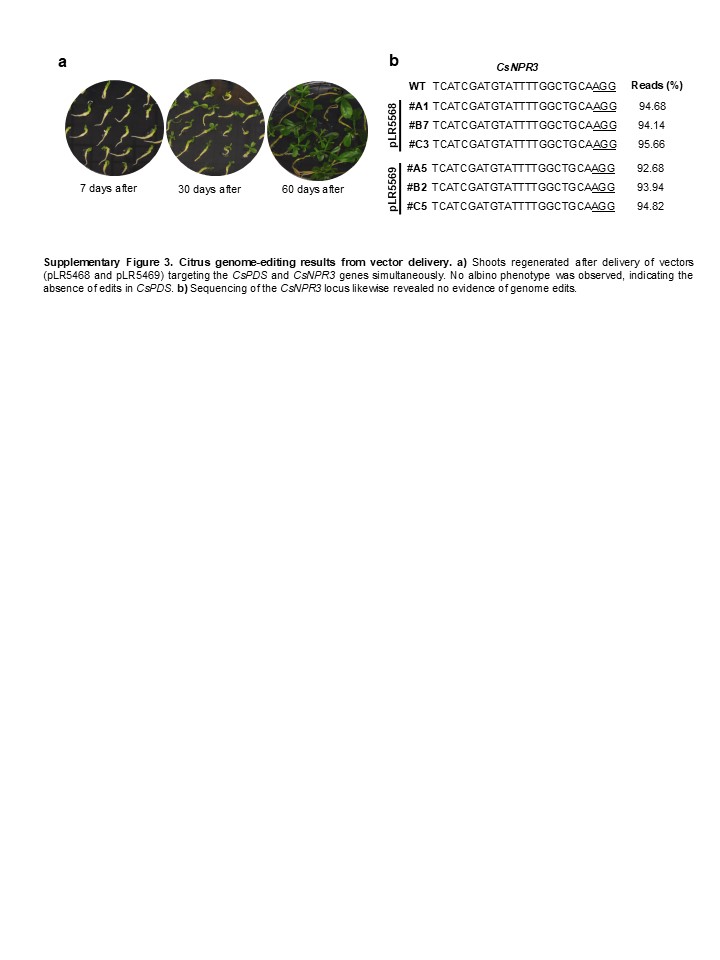


**Supplementary Fig 3. Citrus genome-editing results from vector delivery. (a)** Shoots regenerated after delivery of vectors (pLR5468 and pLR5469) targeting the *CsPDS* and *CsNPR3* genes simultaneously. No albino phenotype was observed, indicating the absence of edits in *CsPDS*. **(b)** Sequencing of the *CsNPR3* locus likewise revealed no evidence of genome edits.


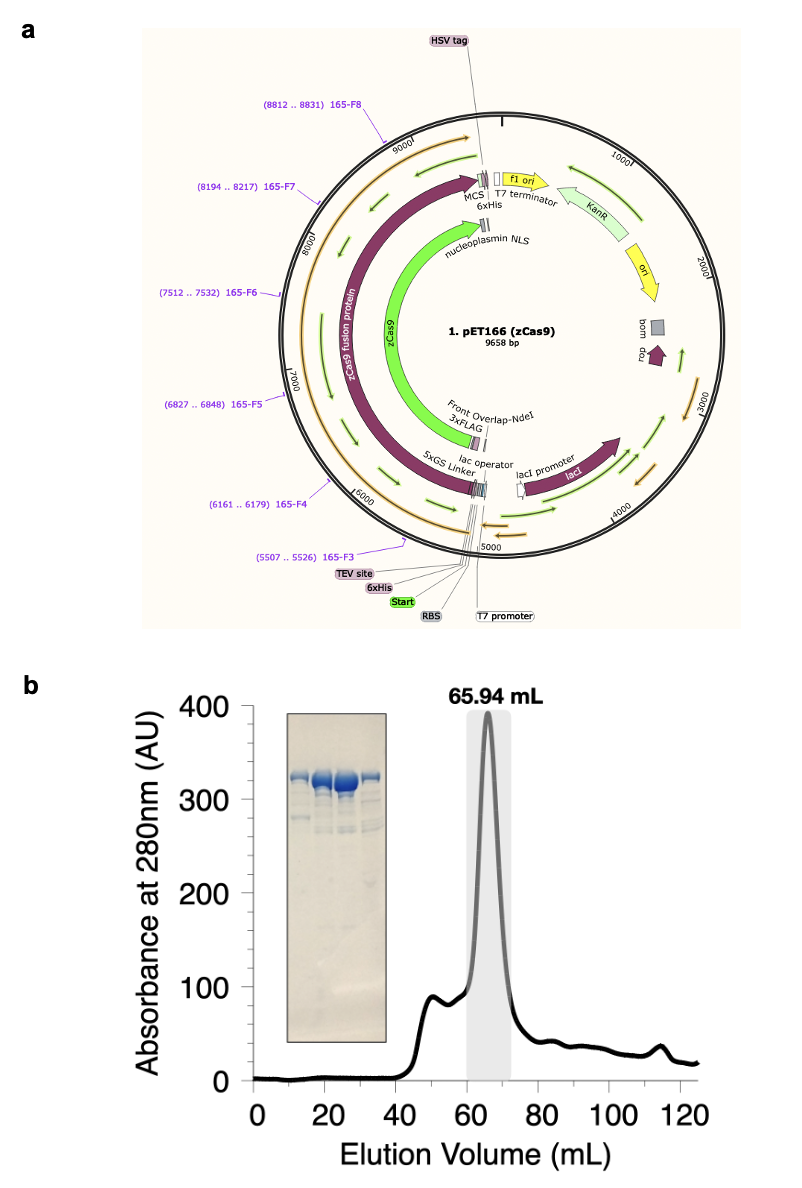


**Supplementary Fig 4. Expression and purification of Cas9 protein.** (a) The Cas9 expression construct was generated by cloning a maize codon–optimized Cas9 (zCas9) gene into the pET27b(+) vector, resulting in pET166. (b) Size-exclusion chromatography elution profile of purified Cas9 obtained using a Cytiva HiLoad 16/600 Superdex 200 column. Inset: SDS–PAGE analysis confirming the purity of Cas9 in the highlighted fractions, which were collected for subsequent genome-editing experiments.

**
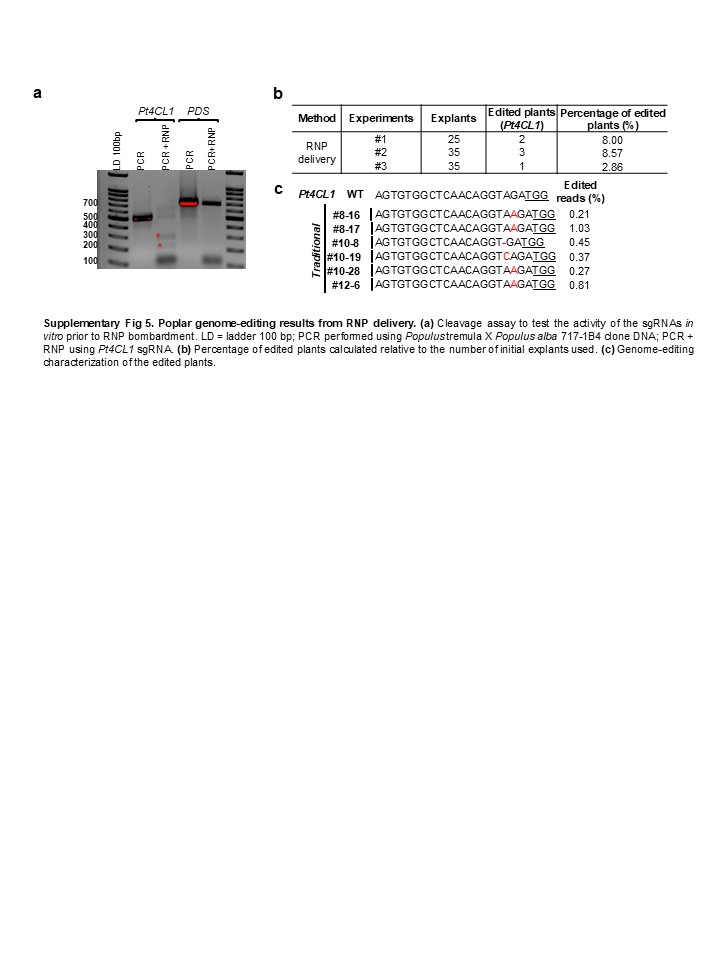
**

**Supplementary Fig 5. Poplar genome-editing results from RNP delivery.** **(a)** Cleavage assay to test the activity of the sgRNAs *in vitro* prior to RNP bombardment. LD = ladder 100 bp; PCR performed using *Populus* tremula X *Populus alba* 717-1B4 clone DNA; PCR + RNP using *Pt4CL1* sgRNA. **(b)** Percentage of edited plants calculated relative to the number of initial explants used. **(c)** Genome-editing characterization of the edited plants.

**Supplementary Table 1. Primers used for Citrus experiments.**

| **Function** | **Primer name** | **Sequence 5'→3'** | **Amplicon (bp)** |
| --- | --- | --- | --- |
| Citrus sgRNA assembly | CsPDS_gR1_F | GGTCAAAAGTTGTAATTGCTGGTGC | **-** |
|  | CsPDS_gR1_R | AAACGCACCAGCAATTACAACTTTT | **-** |
|  | CsNPR3_gRNA1-F | GGTCATCATCGATGTATTTTGGCTGCA | **-** |
|  | CsNPR3_gRNA1-R | AAACTGCAGCCAAAATACATCGATGAT | **-** |
| Citrus vector assembly | Linear_pYPQ166-F1 | CTCAGAGCTTTCGTTCGTAT | 7774 |
|  | Linear_pYPQ166-R1 | TCACTTCTTCTTCTTCGCCT |  |
|  | TLS2-166-F1 | AGGCGAAGAAGAAGAAGTGAATTATCAGAGTGGTGGTGGG | 86 |
|  | TLS2-166-R1 | TACGAACGAAAGCTCTGAGCATCAGAGCGGACCTGTG |  |
|  | Linear_130-F3 | TGGAACTAGAATCTATAATGAGCCT | 3487 |
|  | Linear_130-R3 | ACTCGGTGCCACTTTTTC |  |
|  | TLS2-130-F2 | GAAAAAGTGGCACCGAGTCGGTGCATTATCAGAGTGGTGGTGGG | 306 |
|  | TLS2-130-R2 | AGGCTCATTATAGATTCTAGTTCCA |  |
| Detection of citrus plants transformed with GFP vector and PCR for Sanger sequecing | 131GFP-F3 | ATGTGATATCTCCACTGACGTAAGG | 600 |
|  | 131GFP-R3 | CTGTTGTAGTTGTACTCCAGCTTGT |  |
| Detection of citrus plants transformed with vector pLR5468 | Vector_detection-F | GCAGGCTGAGAATATCATTCATCTCT | 598 |
|  | Vector_detection-R | GAGGCCACGATTTGACACATTTTTAC |  |
| Detection of citrus plants transformed with vector pLR5469 | Vector_detection-F | GCAGGCTGAGAATATCATTCATCTCT | 644 |
|  | Vector_detection-R | GAGGCCACGATTTGACACATTTTTAC |  |
| PCR for the CsPDS sgRNA cleavage assay | CsPDS-PCR-F1 | TTTGTTGCTTTCACTGTTGTCATTG | 720 |
|  | CsPDS-PCR-R1 | ATAACTGTCCATCCACAATGCCATA |  |
| PCR for the CsNPR3 sgRNA cleavage assay | CsNPR3-Check-F | TTACACTGACATGCAACAC | 959 |
|  | CsNPR3-Check-R | CTGGAAAAGTGAAACCAAC |  |
| Hi-TOM primers were used in the first-round PCR for citrus samples | HITOM-CsNPR3-F1 | GGAGTGAGTACGGTGTGCACTCCTGATTGATTCCACTTGTGA | 211 |
|  | HITOM-CsNPR3-F1 | GAGTTGGATGCTGGATGGAAAGGCTTCATATCCAACCTTGC |  |

**Supplementary Table 2. Primers used for Poplar experiments.**

| **Function** | **Primer name** | **Sequence 5'→3'** | **Amplicon (bp)** |
| --- | --- | --- | --- |
| PCR for the PtPDS sgRNA cleavage assay | PtPDS-F2 | GGTATGCAAAGACTTCCTTGC | 704 |
|  | PtPDS-R2 | TCAACACACAGTTTTTTCCC |  |
| PCR for the Pt4CL1 sgRNA cleavage assay | Pt-4CL1-F1 | CCGAGAAAGTGATGTTAAGGTCATG | 506 |
|  | Pt-4CL1-R1 | GAGCCCCTCCAGATTTTATCATCC |  |
| Hi-TOM primers were used in the first-round PCR for poplar samples | HiTOM-Pt4Cl1-F1 | GATGTCGTAGCATTGCCTTATTCATC | 204 |
|  | HiTOM-Pt4Cl1-R1 | CAGACCACAGAGCATTATTGAATTCA |  |

**Supplementary Table 3. sgRNA used for citrus and poplar experiments.**

| **Species** | **Method** | **Gene** | **sgRNA sequence 5'→3'** |
| --- | --- | --- | --- |
| Citrus | Vector delivery | CsPDS | AAAGTTGTAATTGCTGGTGC**AGG** |
|  |  | CsNPR3 | TCATCGATGTATTTTGGCTGCA**AGG** |
|  | RNP delivery | CsPDS | AAAGTTGTAATTGCTGGTGC**AGG** |
|  |  | CsNPR3 | TTGTGTTCATCGATGTATTT**TGG** |
| Poplar | RNP delivery | PtPDS | **CCG**GACCTTGATAACACGGTGAA |
|  |  | Pt4CL1 | AGTGTGGCTCAACAGGTAGA**TGG** |

**References**

Dellaporta, S.L., Wood, J. and Hicks, J.B. (1983) A Plant DNA Minipreparation: Version II. *Plant Molecular Biology Reporter,* 1, 19-21. <https://doi.org/10.1007/BF02712670>

Doyle, J. (1991). DNA Protocols for Plants. In: Hewitt, G.M., Johnston, A.W.B., Young, J.P.W. (eds) Molecular Techniques in Taxonomy. NATO ASI Series, vol 57. Springer, Berlin, Heidelberg. <https://doi.org/10.1007/978-3-642-83962-7_18>

Linsmaier, E. M., & Skoog, F. (1965). Organic growth factor requirements of tobacco tissue cultures. *Physiologia Plantarum*, 18(1), 100–127. <https://doi.org/10.1111/j.1399-3054.1965.tb06874.x>

Liu, Q., Wang, C., Jiao, X., Zhang, H., Song, L., Li, Y., Gao, C., & Wang, K. (2019). Hi-TOM: a platform for high-throughput tracking of mutations induced by CRISPR/Cas systems. *Science China. Life sciences*, *62*(1), 1–7. <https://doi.org/10.1007/s11427-018-9402-9>

Lowder, L. G., Zhang, D., Baltes, N. J., Paul, J. W., 3rd, Tang, X., Zheng, X., Voytas, D. F., Hsieh, T. F., Zhang, Y., & Qi, Y. (2015). A CRISPR/Cas9 Toolbox for Multiplexed Plant Genome Editing and Transcriptional Regulation. *Plant physiology*, *169*(2), 971–985. <https://doi.org/10.1104/pp.15.00636>

Rinne, P. L. H., Paul, L. K., Vahala, J., Ruonala, R., Kangasjärvi, J., & van der Schoot, C. (2015). Long and short photoperiod buds in hybrid aspen share structural development and expression patterns of marker genes. *Journal of Experimental Botany*, 66(21), 6745–6760. <https://doi.org/10.1093/jxb/erv380>

Rocha, D. C., Omoregbee, M. O., Contiliani, D. F., Mandlik, R., Li, G., Mascoveto, J., Coleman, G., Culver, J. N., Leal, D. R., de Souza, A. A., & Qi, Y. (2025). Transgene-free genome editing in citrus and poplar trees using positive and negative selection markers. *Plant cell reports*, *44*(11), 244. <https://doi.org/10.1007/s00299-025-03627-2>

Tang, X., Chen, S., Yu, H., Zheng, X., Zhang, F., Deng, X., & Xu, Q. (2021). Development of a gRNA-tRNA array of CRISPR/Cas9 in combination with grafting technique to improve gene-editing efficiency of sweet orange. *Plant cell reports*, *40*(12), 2453–2456. <https://doi.org/10.1007/s00299-021-02781-7>
